# ALKBH7 Variant Related to Prostate Cancer Exhibits Altered Substrate Binding

**DOI:** 10.1371/journal.pcbi.1005345

**Published:** 2017-02-23

**Authors:** Alice R. Walker, Pavel Silvestrov, Tina A. Müller, Robert H. Podolsky, Gregory Dyson, Robert P. Hausinger, Gerardo Andrés Cisneros

**Affiliations:** 1 Department of Chemistry, Wayne State University, Detroit, MI, United States of America; 2 Department of Microbiology and Molecular Genetics, Michigan State University, East Lansing, MI, United States of America; 3 Wayne State University Department of Family Medicine and Public Health Sciences, Wayne State University, Detroit, MI, United States of America; 4 Karmanos Cancer Institute, Wayne State University, Detroit, MI, United States of America; Baltimore, UNITED STATES

## Abstract

The search for prostate cancer biomarkers has received increased attention and several DNA repair related enzymes have been linked to this dysfunction. Here we report a targeted search for single nucleotide polymorphisms (SNPs) and functional impact characterization of human ALKBH family dioxygenases related to prostate cancer. Our results uncovered a SNP of *ALKBH7*, rs7540, which is associated with prostate cancer disease in a statistically significantly manner in two separate cohorts, and maintained in African American men. Comparisons of molecular dynamics (MD) simulations on the wild-type and variant protein structures indicate that the resulting alteration in the enzyme induces a significant structural change that reduces ALKBH7’s ability to bind its cosubstrate. Experimental spectroscopy studies with purified proteins validate our MD predictions and corroborate the conclusion that this cancer-associated mutation affects productive cosubstrate binding in ALKBH7.

## Author Summary

Improvements in personalized DNA sequencing have led to an increased interest in targeted biomarkers for therapeutic and diagnostic purposes. In this work, we report on a new biomarker for prostate cancer found through a targeted search for single nucleotide polymorphisms (SNPs) of the genes encoding human ALKBH family dioxygenases. Our results uncovered rs7540, which leads to a missense mutation in *ALKBH7*. Comparative molecular dynamics simulations on the wild type and SNP variant of the protein show that the mutation elicits a structural change that dramatically decreases ALKBH7’s affinity for its cosubstrate. This prediction is confirmed by experimental UV-Vis spectroscopy. Taken together, these results give important insights into a novel prostate-cancer related SNP and its impact on the structure and function of ALKBH7.

## Introduction

Prostate cancer is the 2^nd^ leading cause of death from cancer for men[1]. In 2015 the number of new cases in the USA was estimated at 220,800 and the number of deaths from the disease at 27,540[1]. The high prevalence and morbidity of prostate cancer motivates research and development of more efficient methods for preventing and treating this disease. The decreased cost of DNA sequencing and the growing interest in precision medicine have spurred the accumulation of personalized genomic data [2–6]. This development makes it possible to determine mutations of genes that are directly linked to a given phenotype and can aid in the development of novel diagnostic and therapeutic avenues.

DNA undergoes damage from diverse sources, resulting in mutations that can lead to cancer. Cells have a variety of mechanisms to repair damage, and defects in DNA repair and damage response pathways can have deleterious consequences [7]. Indeed, some of these defects have been directly linked to prostate cancer [8,9]. DNA alkylation is a particular type of damage that may result in mutagenic lesions [10]. There are at least three types of DNA repair enzymes to deal with DNA alkylation damage including dioxygenases, glycosylases, and methyltransferases [10]. Some members of the ALKBH family of dioxygenases, named as such because they are homologues of the *Escherichia coli* AlkB DNA repair enzyme, catalyze the oxidation of the alkyl moiety on damaged bases to directly repair the lesion. In contrast, glycosylases, which act on alkylated bases, lead to the formation of abasic sites that require further steps to replace the missing bases. In addition, alkyl lesions can be repaired by specific methyltransferases, but this reaction inactivates the single-use enzyme [11].

There are 9 known homologues of AlkB in humans including ALKBH1 through ALKBH8 and FTO, the fat mass and obesity-associated protein. In all cases the active site consists of two conserved histidines and an aspartate, which coordinate an Fe cation and bind two cosubstrates: α-ketoglutarate (α-kg), and O_2_ [12–16]. The reaction catalyzed by several proteins in the ALKBH family proceeds through an oxidative dealkylation, with the concomitant release of succinate, CO_2_, and the repaired base [14]. The mechanism of AlkB has been extensively studied by experimental and computational means [14,17,18,19]. A more thorough understanding of the structures and functions of the ALKBH enzymes could provide deeper insights into DNA damage repair pathways and allow for the development of more efficient methods for diagnosing or treating diseases. For example, ALKBH2 overexpression has been linked to progression of bladder cancer [20], and ALKBH3 expression contributes to survival of non-small cell lung cancer cells and plays a role in rectal carcinoma [21,22]. Moreover, ALKBH3 is significantly over-expressed in prostate cancer and is also known as prostate cancer antigen-1 (PCA-1) [23].

ALKBH7 is a relatively unstudied member of the ALKBH family. Recently, this protein has been implicated in fat metabolism [24] and programmed necrosis [25]. The latter process performs the important function of eliminating cells that have been too heavily damaged to repair effectively. Specifically, programmed necrosis initiates collapse of the mitochondrial electrochemical potential and, eventually, leads to cell death. Additionally, ALKBH7 is observed to exhibit moderate to strong cytoplasmic immunoreactivity for several cancer phenotypes in the human protein atlas (http://www.proteinatlas.org/ENSG00000125652-ALKBH7/cancer).

The native substrate of ALKBH7 is currently unknown [15], and the protein is missing the characteristic nucleotide recognition lid that would be expected for an enzyme that reacts with nucleic acids [26]. ALKBH7 does, however, have the characteristic, conserved active site residues for ALKBH family members, and it binds catalytically active iron and the α-kg cosubstrate like the rest of the family [15]. This finding suggests that the catalytic activity of ALKBH7 may involve a dealkylation by a hydroxylation process. While AlkB and most ALKBH homologues have a conserved asparagine residue that hydrogen bonds with α-kg, ALKBH7 does not. This change is unique and could indicate a less stable active site than found in the other homologues[15]. Indeed, substitutions of residues in and close to the active site have been shown to disrupt cosubstrate binding in related enzymes [15].

While genome wide association studies (GWAS) will identify some DNA sequence variants linked to a disease, other variants with significant associations to a phenotype may be overlooked due to the large number of variants analyzed and the resulting stringent selection criteria. A recently developed software package called HyDn-SNP-S enables the targeted search of disease-related SNPs to a particular gene or set of genes of interest. This program allows us to search for SNPs of genes encoding DNA repair enzymes with a potential relation to cancer phenotypes [27]. One of the first SNPs found by our software, rs3730477, has recently been experimentally tested for increase in breast cancer risk [28].

Given the relation of some ALKBH family enzymes to prostate cancer, we have performed a targeted search for prostate cancer-related SNPs of *ALKBH* homologues using HyDn-SNP-S, followed by computational and experimental investigations related to a SNP of *ALKBH7* resulting in a missense mutation. Our computational results predict that the resulting ALKBH7 SNP variant exhibits reduced affinity for its cofactor Fe(II) and α-kg or succinate. Ultraviolet-visible (UV-Vis) spectroscopy carried out with the purified proteins confirms these findings.

## Results and Discussion

### SNP discovery and statistical analysis

We used a hypothesis-driven SNP search (HyDn-SNP-S) approach [27] to identify all prostate cancer related SNPs of *ALKBH* genes using data from phs000207.v1.p1 (dbGaP access request #1961) [29]. SNPs having a statistically significant association (p-value < 0.05) with prostate cancer status (case vs. control) were identified in *ALKBH1*, *ALKBH7*, and *FTO* (Table 1, see Supplementary Information S1 Table for results with different genetic models). Subsequently, the dbSNP database of NCBI [30] was employed to determine whether each of the statistically significant SNPs was located in an intronic or exonic region. Our analysis uncovered rs7540 as the only exonic SNP of ALKBH enzymes that is significantly associated with prostate cancer. Using the additive genetic model we computed an odds ratio = 0.81 (0.66, 0.99), p = 0.04. That is, in this cohort composed of European Americans exclusively, this SNP produces a protective phenotype.

**Table 1:** SNPs of *ALKBH* family genes with significant association (p ≤ 0.05) with prostate cancer phenotype.

SNP	Gene	Intronic(I)/ Exonic(E)	Effect of the Missense Mutation on ALKBH7
rs7160307	*ALKBH1*	I	
rs3751812	*FTO*	I	
rs6499653	*FTO*	I	
rs8044769	*FTO*	I	
rs7540	*ALKBH7*	E	Arg to Gln
rs7193938	*FTO*	I	
rs9302652	*FTO*	I	
rs7190492	*FTO*	I	
rs8050136	*FTO*	I	
rs12447481	*FTO*	I	

The rs7540 SNP results in a missense mutation yielding R191Q ALKBH7. To further validate the statistical significance of rs7540, a separate analysis was performed on the phs000306 dataset (see Supplementary Information S1 Table) [31,32]. The significance of SNP rs7540 was maintained in the African American subset (n = 2797) from that cohort, although the direction of the effect flipped. The estimated odds ratio of the Q191 variant (AG) versus the R191 WT protein (GG) (there were no AA’s) was 1.45 (1.01, 2.08), p = 0.046. Thus, for the African American cohort, the odds of having prostate cancer are 45% higher for a man with an AG versus GG genotype for rs7540. The logistic regression model included study identity (this data set consisted of 4 separate studies) as a confounding fixed effect variable. The SNP was not significant for the Latino (p = 0.76) and Japanese (no variation in the SNP) cohorts from the same study.

The fact that rs7540 is associated with prostate cancer status in both cohorts in a statistically significant manner, albeit in opposite directions, is intriguing. Racial differences may explain the disparity as the phs000207 cohort consists of Caucasian individuals exclusively, while phs000306 consists of African American individuals. Other researchers have concluded that allele flipping is not necessarily an error when analyzing heterogeneous populations, such as the ones we studied[33]. The minor allele frequencies observed in the cohorts, 0.088 for phs000207 and 0.023 for phs000306, are consistent with the information available in dbSNP for the 2 ethnic groups. A non-wildtype genotype for rs7540 is observed in 16.5% of the Caucasian population and in 2.3% of the African American population.

### Computational results

We performed MD simulations on four different systems to investigate the functional impact of the rs7540 SNP on the encoded protein; i.e., the wild-type (WT) enzyme and the R191Q ALKBH7 variant, with either α-kg or succinate in the active site. Each model was simulated for 500 ns (in triplicate, 1.5 μs total aggregate simulation time for each system) as described in the Methods section. Comparison of the simulation results for native enzyme and the variant encoded by the SNP mutant revealed striking differences, regardless of whether α-kg or succinate was included in the active site. Namely, the R191Q variant results in the removal of a key hydrogen bond, located ca. 22 Å away from the active site. This change does not significantly affect the overall protein structure (see Supplementary Information S1 Fig and S2 Table). However, the R191Q substitution produces a conformational change that is transmitted to the active site, and results in the reduction in binding affinity for α-kg or succinate (see below).

**Fig 1:**
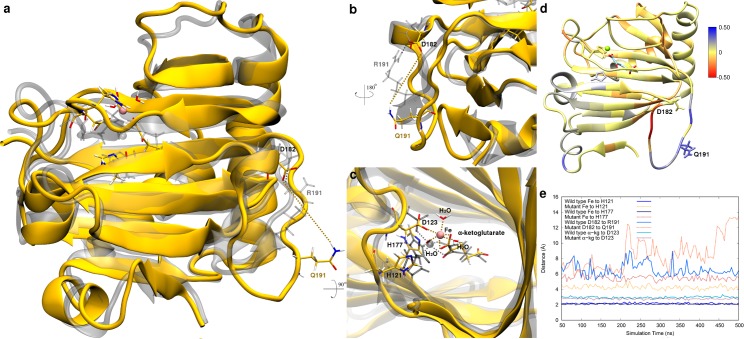
Structural and dynamic comparison between WT and R191Q ALKBH7 with bound α-kg. **a,** Overlay of representative structures for WT (gray) and R191Q mutant (yellow) forms of ALKBH7. Active site residues and α-kg as well as the site undergoing substitution are displayed (licorice). **b,** 180 degree rotation and close-up of the substituted site. **c,** 90 degree rotation and close-up of the active site, with each relevant active site residue and α-kg labeled. Dashed lines in gray represent the original bonds to the metal ion in the crystal structure, and dashed lines in orange represent the new bonds to the metal ion near the end of the trajectory for the variant protein. **d,** Correlation difference for each residue in the WT protein with respect to the R191Q variant mapped onto the protein structure using the mutation site as the reference. **e,** Distance analysis for key residues in the SNP variant and active sites (with respect to their centers of mass) throughout the simulation trajectory.

**Fig 2:**
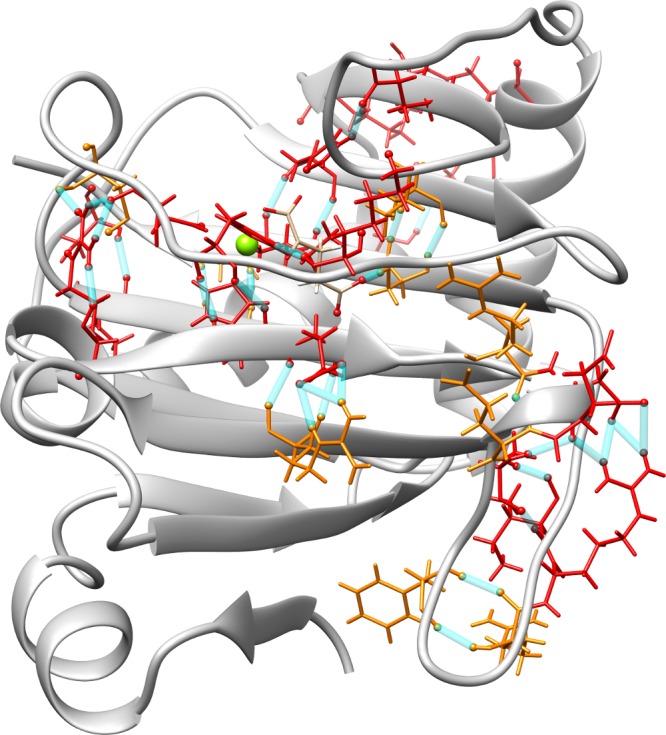
Hydrogen bond analysis for the WT/R191Q variant with α-kg. Residues colored in red denote amino acids involved in H-bonds for over 30% of the WT trajectory and broken for over 90% of the R191Q variant trajectory. Residues colored in orange are involved in hydrogen bonds for both trajectories, but are present for at least 30% less of the time in the variant trajectory. The hydrogen bonds between these residues are displayed in blue. The corresponding analysis for the WT/SNP variant with succinate are given in the Supplementary Information.

The mutation that generates ALKBH7 with R191 changed to Q removes a key H-bond between residue 191 and D182 regardless of whether the active site is occupied by the cosubstrate (α-kg), as shown in Fig 1A, or by the product (Supplementary Information S2 Fig). This modification of the protein results in several structural and dynamic changes including the loss of a β-hairpin at the substituted site (Fig 1B) and changes in hydrogen bonding patterns (Fig 2 and Supplementary Information S3 Fig and S4 Fig). The removal of the key H-bond between R191 and D182 produces a series of structural changes that are transmitted to the active site and result in significant rearrangement of the key residues that bind the catalytic cation (Fig 1C). Moreover, the substitution associated with the rs7540 SNP also affects the motion of the protein in the substituted site and several β-strands that form the “jelly-roll” fold of the protein (Fig 1B and 1D). The MD simulations clearly show that the coordination shell of the cation in the active site of the WT structure is stable as evidenced by the unchanging distances of the coordinating His residues to the Fe(II), whereas these distances are significantly affected in the R191Q variant (Fig 1E and 1C).

Fig 2 highlights some of the largest changes in hydrogen bonding between the WT and R191Q variants. The largest shifts in hydrogen bonding are present at the site of the mutation, as one would expect, but also in and around the active site. The fact that most of the broken hydrogen bonds are broken for at least 90% of the mutant trajectory also indicates that these are not transient changes in structure, but rather permanent shifts induced by the mutation. Additionally, a great majority of the hydrogen bonds outside of those two areas remain completely intact, which correlates with the low RMSD for the protein backbone for both the WT and R191Q variant (S1 Fig, S2 Table). On average, the low RMSD for all trajectories (≈1.6±0.3 Å on average) indicates relatively stable structures despite the shifts in hydrogen bonding.

**Fig 3:**
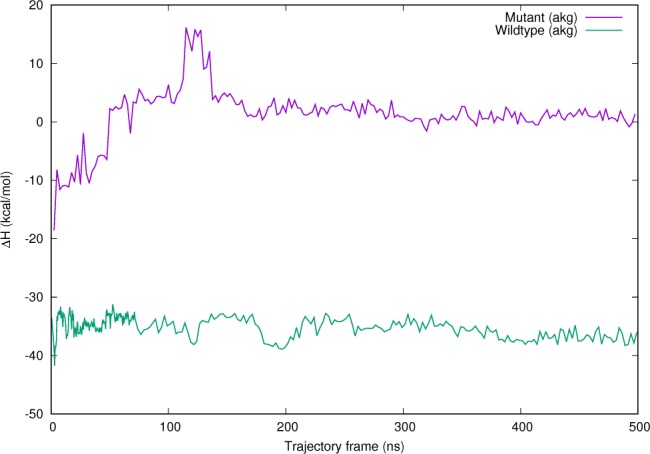
Average binding enthalpies for cosubstrate α-kg and Fe at the active site (in kcal/mol) for the simulation. Results for the product (succinate) are reported in the Supplementary Information.

In addition to the structural changes, we performed a binding analysis of the Fe-α-kg and Fe-succinate complexes to the protein using MMPBSA as described in the Methods section. A significant decrease in binding enthalpy (Δ*H*_bind_) and free energy (Δ*G*_bind_) is observed in the R191Q variant compared to the native structure regardless of whether the active site contains the cosubstrate or the product (≈30±4 kcal/mol). Interestingly, the simulation for the R191Q variant structure with both α-kg (Fig 3) and succinate (Supplementary Information S5 Fig) revealed changes in the binding enthalpy compared to the WT structure in the time-scale of the simulation. Taken together, these results strongly point to a structural and dynamic variation in the R191Q protein that has a drastically different binding affinity for Fe-α-kg and Fe-succinate cofactors.

### UV-Vis spectroscopy

**Fig 4:**
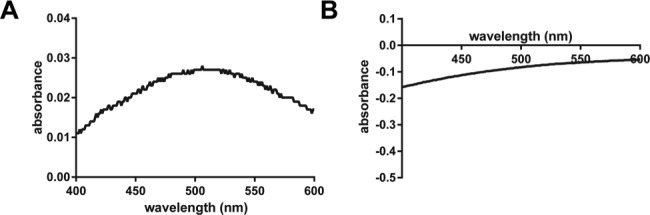
Difference absorption spectra of WT ALKBH7 and its R191Q variant. The spectra of the anaerobic proteins (0.3 mM) were recorded in the presence of 2 mM α-kg and 100 μM Fe(II). The difference spectra were obtained by subtracting the spectra for proteins with α-kg, but without the metal. **A**, WT ALKBH7; **B**, R191Q ALKBH7.

Based on our MD simulation predictions of altered α-kg or succinate binding to the R191Q variant of ALKBH7, we directly tested for such a perturbation of the active site by examining whether the variant protein possessed a diagnostic spectroscopic feature observed in α-kg-dependent oxygenases. Members of this class of enzymes form weak metal-to-ligand charge transfer (MLCT) electronic transitions at 500–530 nm (Δε 140–270 M^-1^ cm^-1^) in the presence of α-kg and Fe(II) under anaerobic conditions [34–37]. The WT ALKBH7 enzyme and the R191Q variant were overproduced in *E*. *coli* and purified (Supplementary Information S6 Fig), then difference UV-Vis spectroscopy of the anaerobic proteins was performed. The spectrum of WT ALKBH7·Fe(II)·α-kg minus that of ALKBH7·α-kg revealed the typical weak MLCT transitions with a maximum at 510 nm (Fig 4). Substoichiometric iron concentrations were purposely used to assess chromophore stability, so the apparent Δε_510_ of ~100 M^-1^ cm^-1^ is consistent with results previously reported for other family members. In contrast, the difference spectrum of the R191Q variant did not exhibit this spectroscopic feature. Furthermore, the variant protein was unstable during the spectroscopic study leading to partial precipitation of the protein and resulting in a negative absorption in the difference spectrum. These findings were observed in duplicate preparations of the protein species, and they support the interpretations from computational simulations that suggest that the active site of the R191Q variant of ALKBH7 is structurally different from that in WT enzyme.

Whereas the WT ALKBH7·Fe(II)·α-kg species exhibited MLCT transitions that are the hallmark of this family of enzymes [36,37], no similar increase in absorption was seen with the R191Q variant in our UV-Vis spectroscopy studies. This result confirms the finding of MD simulations and provides evidence that the active site of the R191Q variant is structurally different from that of the WT ALKBH7, with less tight binding of the divalent metal cofactor and the α-kg cosubstrate.

Taken together, our findings show that rs7540 is indicative of prostate cancer in a statistically significant manner. The difference in the effect of rs7540 on the two ethnically diverse cohorts is intriguing and should prompt further investigation in ethnically diverse cell lines. Our combined experimental and computational results show the R191Q variant cannot bind the co-factor/substrate, which may impair its enzymatic activity, and, in turn, could be a contributor to the predisposition for prostate cancer in African American individuals harboring this particular SNP. In addition to being deficient in binding Fe(II) and α-kg, the variant was generally less stable than the WT protein as demonstrated by its propensity to precipitate. These findings indicate that the targeted single amino acid change has a profound impact on the protein structure and the behavior of this variant might limit its function *in vivo* both due to structural changes at the active site and to being less stable.

In sum, a targeted search of prostate cancer-related SNPs on *ALKBH* family genes revealed a single exonic nonsynonymous SNP, rs7540, on *ALKBH7*. This SNP results in a missense mutation, which leads to the R191Q substitution in the translated protein. This SNP is associated with prostate cancer disease in a statistically significant manner (validated on two different prostate cancer data bases). MD simulations indicate that the amino acid substitution induces structural changes that affect the ability of ALKBH7 to bind its catalytic cation and cosubstrate (or product) without a gross overall change of the protein tertiary structure. Experimental UV-Vis spectroscopy validated the computational predictions and showed the prostate cancer-associated R191Q variant had a profound reduction in the binding affinity for its cation and cosubstrate. In addition to the functional impact, this sequence difference may provide a possible target for diagnostic and/or therapeutic purposes.

## Methods

### SNP discovery and statistical analysis

Two case/control GWAS analyses from the dbGaP [38] were employed for the search of SNPs and/or haplotypes (access request #1961). The initial search was performed on the data from phs000207.v1.p1[29], which included 1,172 individuals with prostate cancer and 1,157 controls of European descent. Hypothesis driven analysis was performed with a focus on finding associations between the mutations in genes from the ALKBH family (ALKBH1 through ALKBH8 and FTO) and prostate cancer. SNPs located in the genes of interest were identified using the hypothesis driven SNP search (HyDn-SNP-S) program [27]. Only SNPs located in the genes of interest were analyzed using logistic regression models in R to evaluate the association between SNP and case/control status. Different genetic inheritance models were considered (additive, dominant, recessive, and multiplicative). A literature search was performed to identify those SNPs that could be mapped to available crystal structures of the proteins of interest. Once the SNP list was obtained, we statistically validated the SNP association with cancer risk using the same analysis on a second prostate cancer database, phs000306, which consisted of 1423 and 1373 African American cases and control, respectively [31,32].

### Computational simulations

The initial crystal structure (pdbid 4QKD) [15] contained one missing alanine residue (A100) that was introduced using Modeller [39], with all other residues restrained, and subsequently checked and hydrogenated using MolProbity [40]. Four systems were created to determine the impact of the mutation from the rs7540 SNP; two WT structures [15] and two R191Q mutant structures with either α-kg or succinate bound at the metallocenter. All MD simulations were performed in triplicate with the starting structure for each system taken from the initial WT trajectory. For the mutant structures, the chosen snapshots were modified by performing the amino acid substitution and/or substrate/product replacement as necessary. Key snapshots along the mutant protein trajectories were targeted for further dynamic simulations as well. In total, 12 simulations were run with 3 unique starting points per system for at least 500 ns each.

All simulations were performed with the pmemd.cuda program from AMBER14 [41,42] using the ff99SB force field [43] with a 1 fs timestep. An 8 Å cutoff was used for all nonbonded interactions, and sPME for long-range electrostatics [44]. SHAKE was applied to all bonds involving hydrogen atoms. All protein structures were solvated in a rectangular box of TIP3P water [45] using a 12 Å pad between the surface of the protein and the edge of the box. The cation for all systems was simulated using Mg^2+^ as an appropriate surrogate [46]. All runs were done in the NVT ensemble; the initial thermalization and equilibration was performed with the Berendsen thermostat [47], followed by NVT calculations using the Langevin thermostat [48] for production, with a heat bath coupling constant of 1 fs. The initial and final configurations, and selected snapshots along the simulations, were used for non-covalent interaction (NCI) analysis (see SI for description) [49].

The MMGBSA module of the MMPBSA.py program [50,51] in AMBER14 was used to calculate the binding affinities of the metal-cofactor complex to the active site of the protein. Energies were calculated using the Generalized Born implicit solvent model [52], with an approximate quasi-harmonic entropy calculation using the default MMGBSA parameters. Individual energy calculations were performed on sets of 200 protein-ligand configurations taken in 2.5 ns increments along each explicit solvent trajectory in order to obtain qualitative relative binding affinities (50,000 configurations calculated per trajectory). To replace the stripped counterions, an additional salt concentration parameter was set to 0.10 M.

### Experimental methods

The plasmid encoding a truncated ALKBH7 with an *N*-terminal His-tag was provided by Dr. Chen (Beijing, China) [15]. The R191Q variant was created using the QuickChange site directed mutagenesis protocol (Qiagen) according to the manufacturer’s instructions with primers 5'-CTTTGGGGAACGCCAGATTCCCCGGGGC-3' and 5'-GCCCCGGGGAATCTGGCGTTCCCCAAAG-3'. The presence of the mutation was confirmed by sequencing.

His-tagged ALKBH7 and its R191Q variant were overproduced in *E*. *coli* BL21(DE3) cells by methods that were described earlier [35]. Cell-free extract preparation and protein purification is described in detail in the supplementary information.

UV-Vis spectroscopy was carried out using a Shimadzu UV-2600 by procedures that have been previously described [35]. Briefly, all stock solutions, the assay buffer, and WT ALKBH7 and the variant protein were made anaerobic by using a Schlenk line to carry out several rounds of degassing with vacuum and flushing with argon gas. To remove any traces of oxygen, Na_2_S_2_O_4_ was added to the assay buffer to a final concentration of 0.5 mM. All spectroscopic assays were carried out in an anaerobic quartz cuvette containing assay buffer, 0.3 mM protein, and 0.2 mM α-kg. For each sample, the cuvette was centrifuged for 1 min at 3234 *g* before the spectrum was recorded. Fe(NH_4_)_2_(SO_4_)_2_ was added to a final concentration of 100 μM, the cuvette was centrifuged as before, and the spectrum was again obtained. Difference spectra were calculated by subtracting the spectra obtained for WT and variant proteins with α-kg from the spectra for proteins, α-kg, and 100 μM Fe(II).

## Supporting Information

S1 Text Supporting Information. Detailed methods section, additional structural, dynamical and binding analysis as well as SDS-PAGE analysis.
(PDF)

S1 Fig Backbone root mean squared deviation (RMSD) for all four tested systems (WT with α-kg and succinate (suc), and the R191Q mutant with α-kg and succinate). (TIF)

S2 Fig Structural and dynamic comparison between WT and R191Q ALKBH7 with bound succinate. **a**, Overlay of representative structures for WT (gray) and R191Q variant (blue) forms of ALKBH7. Active site residues and succinate as well as the site undergoing substitution are displayed (licorice). **b**, 180 degree rotation and close-up of the substituted site. **c**, 90 degree rotation and close-up of the active site, with each relevant active site residue and succinate labeled. Dashed lines in gray represent the original bonds to the metal ion in the crystal structure, and dashed lines in blue represent the new bonds to the metal ion near the end of the trajectory for the mutant protein. **d**, Correlation difference for each residue in the WT protein with respect to the R191Q variant mapped onto the protein structure using the substituted site as the reference. **e**, Distance analysis for key residues in the mutation and active sites (with respect to their centers of mass) throughout the simulation trajectory.
(TIF)

S3 Fig Hydrogen bond analysis for the tested systems. Residues colored in red denote amino acids involved in H-bonds for over 30% of the WT trajectory and broken for over 90% of the R191Q variant trajectory. Residues colored in orange are involved in hydrogen bonds for both trajectories, but are present for at least 30% less of the time in the variant trajectory. The hydrogen bonds between these residues are displayed in blue. This figure is for the WT/R191Q variant with succinate.
(TIF)

S4 Fig **NCI plots of WT (a-d) and R191Q variant (e-h) ALKBH7.** Panels show representative structures at different stages of the simulation showing the points prior to (a and e), during (b, c, f, and g) and after (d and h) the structural transition. The H-bonds between R191 and D182 in the WT structure that are removed in the SNP variant are circled in black (c).
(TIF)

S5 Fig Average binding enthalpies for product succinate and Fe at the active site (in kcal/mol) over 500ns. (TIF)

S6 Fig SDS-PAGE analysis of ALKBH7. His-tagged WT ALKBH7 and its R191Q variant were purified by using a Ni-NTA Sepharose column, treated with TEV protease to remove the His-tag, and rechromatographed on the Ni-NTA Sepharose column to obtain the non-tagged proteins. The purified and concentrated proteins were analyzed by SDS-PAGE. Lane 1, WT ALKBH7; lane 2, R191Q ALKBH7; lane 3, His-tagged R191Q ALKBH7.
(TIF)

S1 Video Animation of the overall change in the mutant structure with succinate cofactor over the course of a representative simulation, with zooms of the active site (top right) and mutation site (bottom right). Each panel has NCIplot surfaces to demonstrate the change in the intermolecular forces, updated at key points along the animation.
(GIF)

S1 Table SNPs with significant association to a prostate cancer phenotype. (DOCX)

S2 Table Average distances and structural details across all duplicate trajectories. The average distances, RMSD of the protein backbone, and the total number of hydrogen bonds over the entirety of each trajectory were calculated individually and then averaged together to obtain the average and standard deviation values across trajectories so that the replicate runs could be compared.
(DOCX)

S3 Table Average change in binding affinities between the duplicate trajectories. Energy for the metal-cofactor complex before and after the conformational shift (Δ*G*_*shift*_*)*, average free energy of binding for the cofactor-metal complex (Δ*G*_*binding*_*)*, average change in binding enthalpy for the metal-cofactor complex before and after the conformational shift (ΔH_*shift*_) and the average enthalpy of binding for the cofactor-metal complex (Δ*H*_*binding*_) between the duplicate trajectories. All energies are listed in kcal/mol.
(DOCX)
