# Supplementary material for: ALKBH7 Variant Related to Prostate Cancer Exhibits Altered Substrate Binding
Source: PLoS Comput Biol. 2017 Feb 23;13(2):e1005345. doi: 10.1371/journal.pcbi.1005345 (PMC5322872; doi:10.1371/journal.pcbi.1005345)
Supplement: S1 Text — Detailed methods section, additional structural, dynamical and binding analysis as well as SDS-PAGE analysis. (PDF) [file pcbi.1005345.s001.pdf]

Supplementary Information for:

AlkBH7 Variant Related to Prostate Cancer Exhibits Altered Substrate Binding

Alice R. Walker[1], Pavel Silvestrov[1], Tina A. Müller[2], Robert H. Podolski[3], Gregory Dyson[4], Robert P. Hausinger[2], G. Andrés Cisneros[1,4]

[1]Department of Chemistry, Wayne State University, Detroit, MI 48202
[2]Department of Microbiology and Molecular Biology, Michigan State University, East Lansing, MI 48824
[3]Wayne State University Department of Family Medicine and Public Health Sciences, Detroit, MI, 48202
[4]Karmanos Cancer Institute, Wayne State University, Detroit, MI 48202

Detailed Methods

NCIplot

Non-covalent interaction (NCI)[1] analysis plots were generated for the beginning and ending configurations as well as key points along the trajectories. This approach provides a visualization tool that allows the analysis of noncovalent interactions by plotting the reduced electron density gradient versus the sign of the second value of the electron density Hessian matrix, sign(λ_2), times the electron density, ρ. These calculations give qualitative information about the interactions; specifically, the sign(λ_2) corresponds to the type of interaction and the ρ corresponds to the strength. For convenience, the surfaces are color-coded depending on the nature of the interaction: large negative values denote strongly attractive forces, such as hydrogen bonds, and are colored blue, large positive values denote strongly repulsive values, e.g. steric strain, and are colored red, and small values that can be positive or negative denote weak forces, such as van der Waals interactions, which are colored green. 10Å spheres of protein and solvent structures centered on either the Fe atom or on residue 191 were used to calculate the intermolecular interactions, with Fe or residue 191 defined as the ligand. All other parameters thickness of border around the default cube and cube grid limits were set as the default 2 bohr and 0.1/0.1/0.1 Å respectively.

Protein purification

His-tagged ALKBH7 and its R191Q variant were overproduced in *Escherichia coli* BL21(DE3) cells by methods that were described earlier [2]. To prepare cell-free extracts, the harvested cells were resuspended in lysis buffer (20 mM Tris, pH 8, containing 0.3 M NaCl, 1 mM 2-mercaptoethanol, 0.1% Triton X-100, and 0.2 mM phenylmethylsulfonylfluoride), sonicated, and centrifuged at 100,000 *g* for 45 min at 4 ˚C to remove membranes and cell debris. Cell-free extracts were loaded onto a Ni-nitrilotriacetic acid (NTA) Sepharose column (1.5 x 4.5 cm) that had been equilibrated in buffer A (20 mM Tris, pH 8, containing 0.3 M NaCl, 1 mM 2-mercaptoethanol, 10 mM imidazole, and 5% glycerol), the column was washed with three column volumes of buffer A, and the His-tagged proteins were eluted in buffer A containing 200 mM imidazole. Fractions were analyzed by sodium dodecyl sulfate (SDS)-polyacrylamide gel electrophoresis (PAGE) for the presence of ALKBH7. The appropriate fractions were pooled and incubated overnight with His-tagged tobacco-etch virus (TEV) protease [3] at a 1:10 (protease:ALKBH7) weight ratio while dialyzing into buffer A at 4 ˚C. The samples were re-loaded onto the Ni-NTA Sepharose column to remove the TEV protease, the His-tag, and uncleaved protein. The flow-through fractions were collected,

ethylenediaminetetraacetic acid (EDTA) was added to a final concentration of 1 mM and the proteins were concentrated by using Amicon Ultra Centrifugal Filters (Millipore). Prior to UV-Vis spectroscopy experiments, the proteins were dialyzed overnight into assay buffer (20 mM 4-(2-hydroxyethyl)-1-piperazineethanesulfonic acid (Hepes), pH 8, containing 0.1 M NaCl) at 4 ˚C.

SNP Discovery

S1 Table. SNPs with significant association to a prostate cancer phenotype

#	SNP	Gene	p-value	Intron/ Exon	Effects of the Missense Mutation
	**Multiplicative Model**				
1	rs7160307	*ALKBH1*	0.00754	I	
2	rs3751812	*FTO*	0.04365	I	
3	rs6499653	*FTO*	0.029584	I	
4	rs8044769	*FTO*	0.048004	I	
	**Recessive Model**				
1	rs7160307	*ALKBH1*	0.044301	I	
2	rs7540	*ALKBH7*	0.029142	E	Arg to Gln
3	rs6499653	*FTO*	0.009707	I	
4	rs7193938	*FTO*	0.020805	I	
5	rs8044769	*FTO*	0.014301	I	
6	rs9302652	*FTO*	0.034896	I	
	**Additive Model**				
1	rs7160307	*ALKBH1*	0.012976	I	
2	rs7540	*ALKBH7*	0.042607	E	Arg to Gln
3	rs3751812	*FTO*	0.018385	I	
4	rs6499653	*FTO*	0.00888	I	
5	rs7190492	*FTO*	0.035297	I	
6	rs7193938	*FTO*	0.038663	I	
7	rs8044769	*FTO*	0.030062	I	
8	rs8050136	*FTO*	0.022015	I	
9	rs9302652	*FTO*	0.031647	I	
	**Dominant Model**				
1	rs7160307	*ALKBH1*	0.006068	I	
2	rs3751812	*FTO*	0.019815	I	
3	rs8050136	*FTO*	0.022225	I	
4	rs12447481	*FTO*	0.033246	I	

MD simulations

S1 Figure: Backbone root mean squared deviation (RMSD) for all four tested systems (WT with α-kg and succinate (suc), and the R191Q mutant with α-kg and succinate).

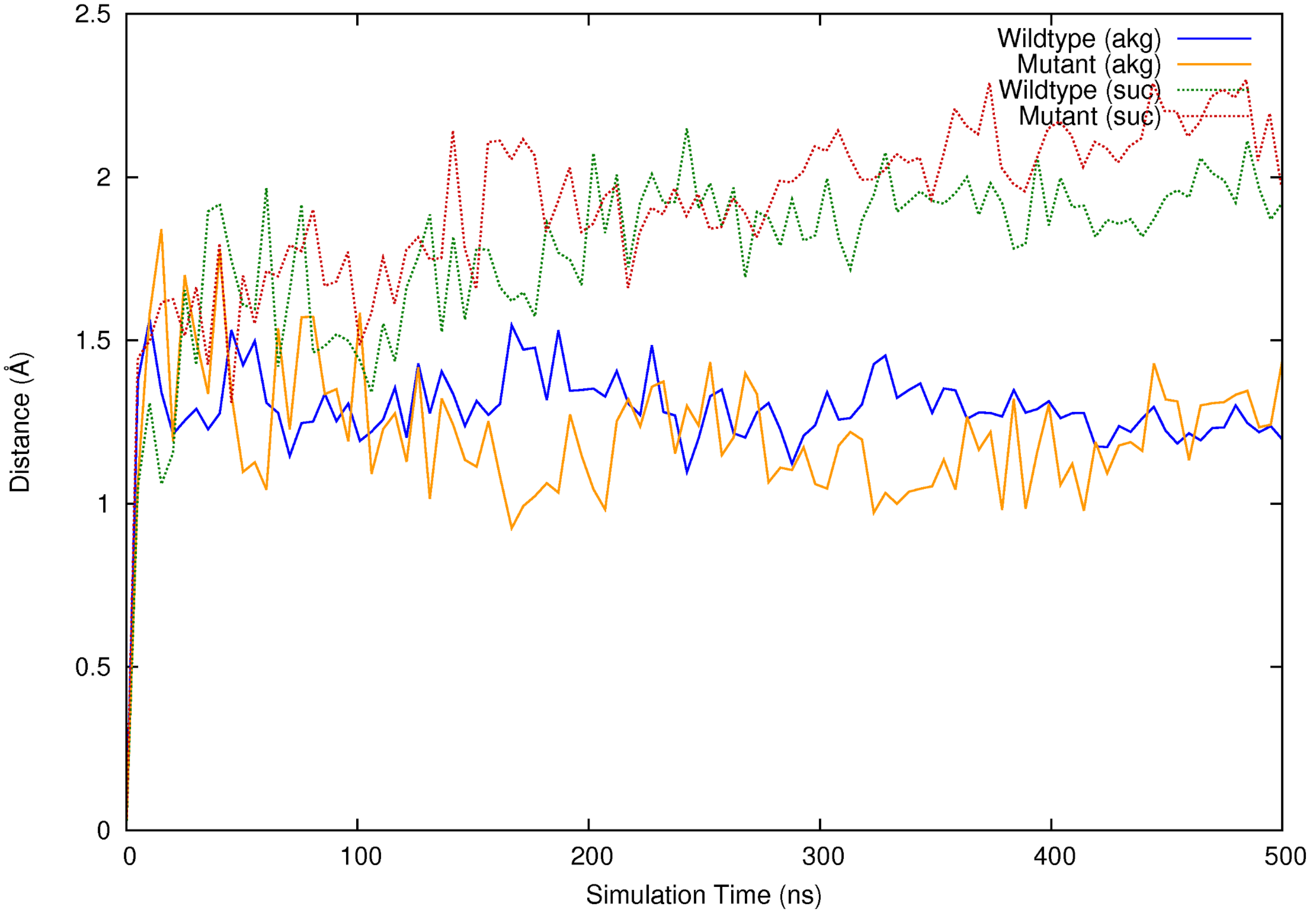

S2 Figure: Structural and dynamic comparison between WT and R191Q ALKBH7 with bound succinate. **a**, Overlay of representative structures for WT (gray) and R191Q variant (blue) forms of ALKBH7. Active site residues and succinate as well as the site undergoing substitution are displayed (licorice). **b**, 180 degree rotation and close-up of the substituted site. **c**, 90 degree rotation and close-up of the active site, with each relevant active site residue and succinate labeled. Dashed lines in gray represent the original bonds to the metal ion in the crystal structure, and dashed lines in orange represent the new bonds to the metal ion near the end of the trajectory for the mutant protein. **d**, Correlation difference for each residue in the WT protein with respect to the R191Q variant mapped onto the protein structure using the substituted site as the reference. **e**, Distance analysis for key residues in the mutation and active sites (with respect to their centers of mass) throughout the simulation trajectory.

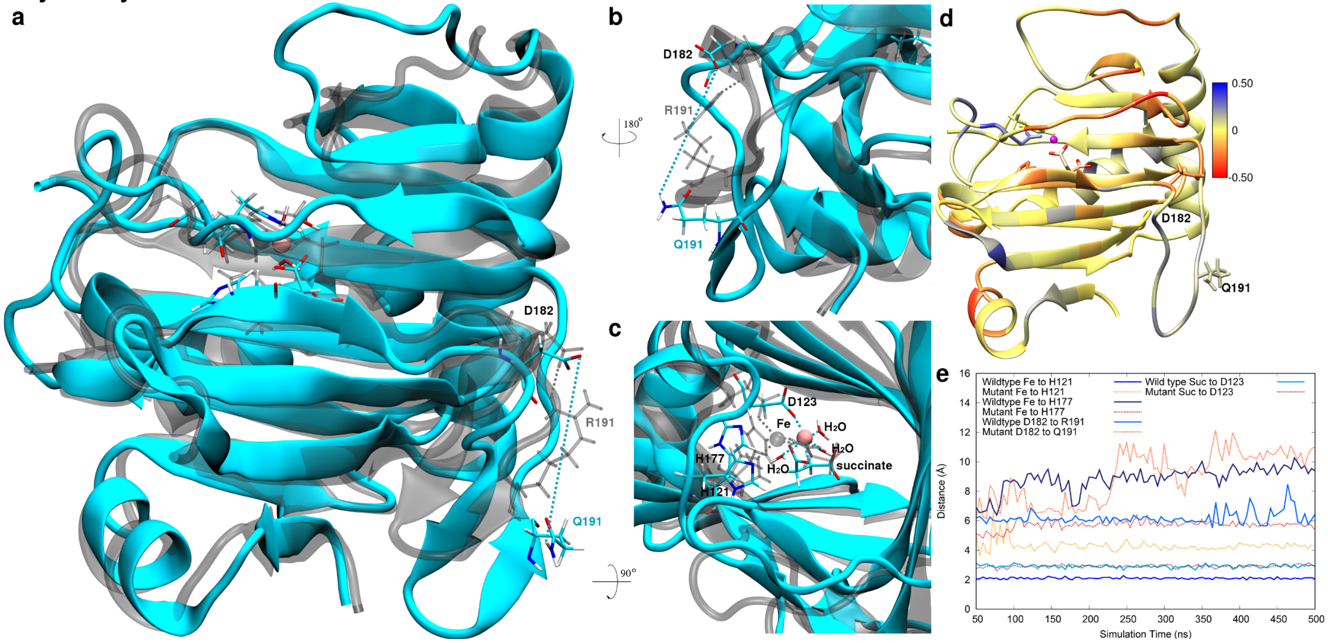

S2 Table: Average distances and structural details across all duplicate trajectories. The average distances, RMSD of the protein backbone, and the total number of hydrogen bonds over the entirety of each trajectory were calculated individually and then averaged together to obtain the average and standard deviation values across trajectories so that the replicate runs could be compared.

Trajectory type	Fe to H121(Å)	Fe to H177(Å)	D182 to R191/Q191(Å)	Suc or akg to D123(Å)	Backbone RMSD (Å)	Total # hydrogen bonds
Wild type	2.10±0.05	4.05±2.81	6.30±0.39	3.02±0.27	1.52±0.20	112±4
Mutant	4.14±0.03	5.59±0.46	8.03±0.99	2.82±0.10	1.68±0.44	107±3

S3 Figure: Hydrogen bond analysis for the tested systems. Residues colored in red denote amino acids involved in H-bonds for over 30% of the WT trajectory and broken for over 90% of the R191Q variant trajectory. Residues colored in orange are involved in hydrogen bonds for both trajectories, but are present for at least 30% less of the time in the variant trajectory. The hydrogen bonds between these residues are displayed in blue. This figure is for the WT/R191Q variant with succinate.

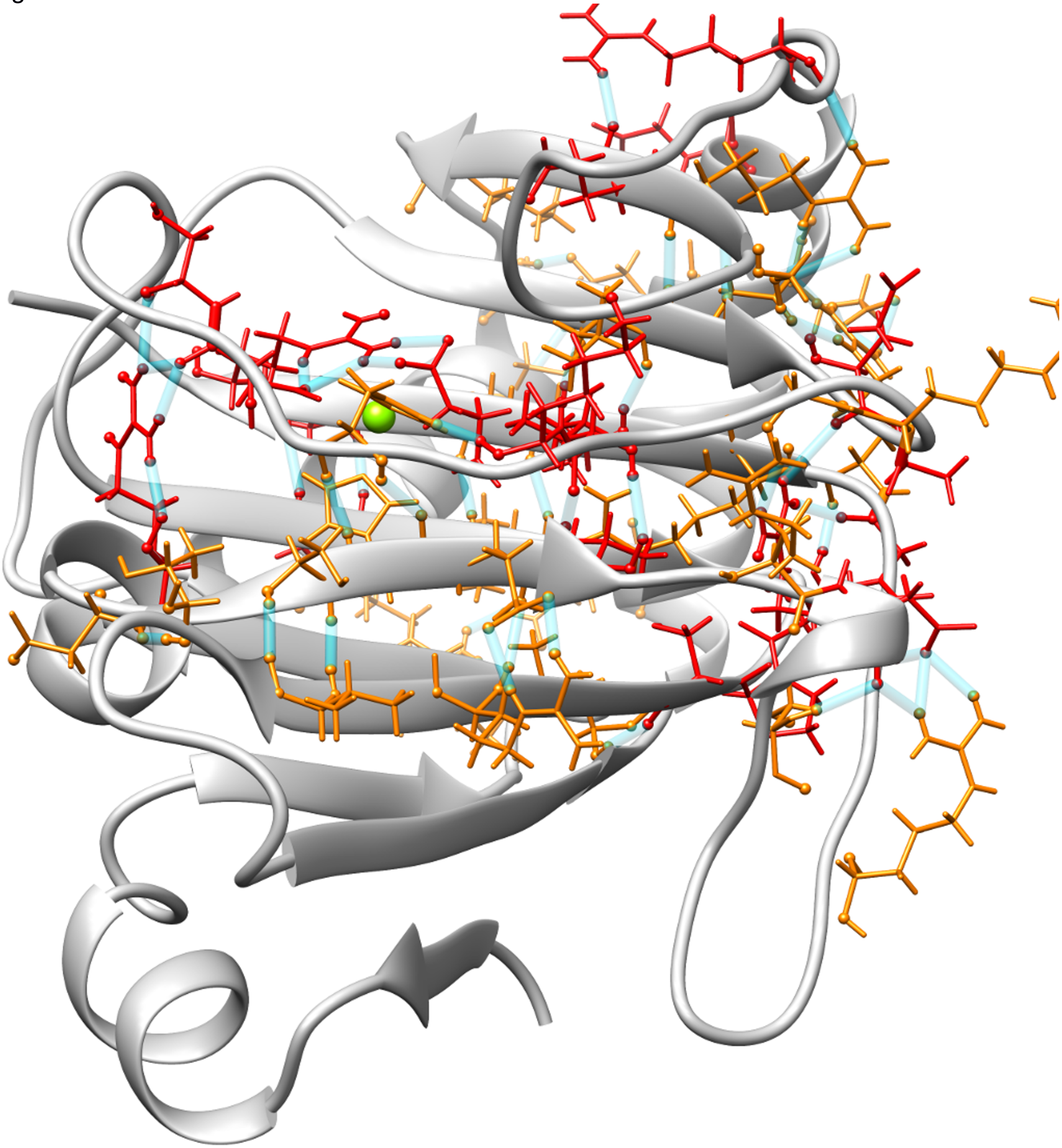

S4 Figure: NCI plots of WT (a-d) and R191Q variant (e-h) ALKBH7. Panels show representative structures at different stages of the simulation showing the points prior to (a and e), during (b, c, f, and g) and after (d and h) the structural transition. The H-bonds between R191 and D182 in the WT structure that are removed in the SNP variant are circled in black (c).

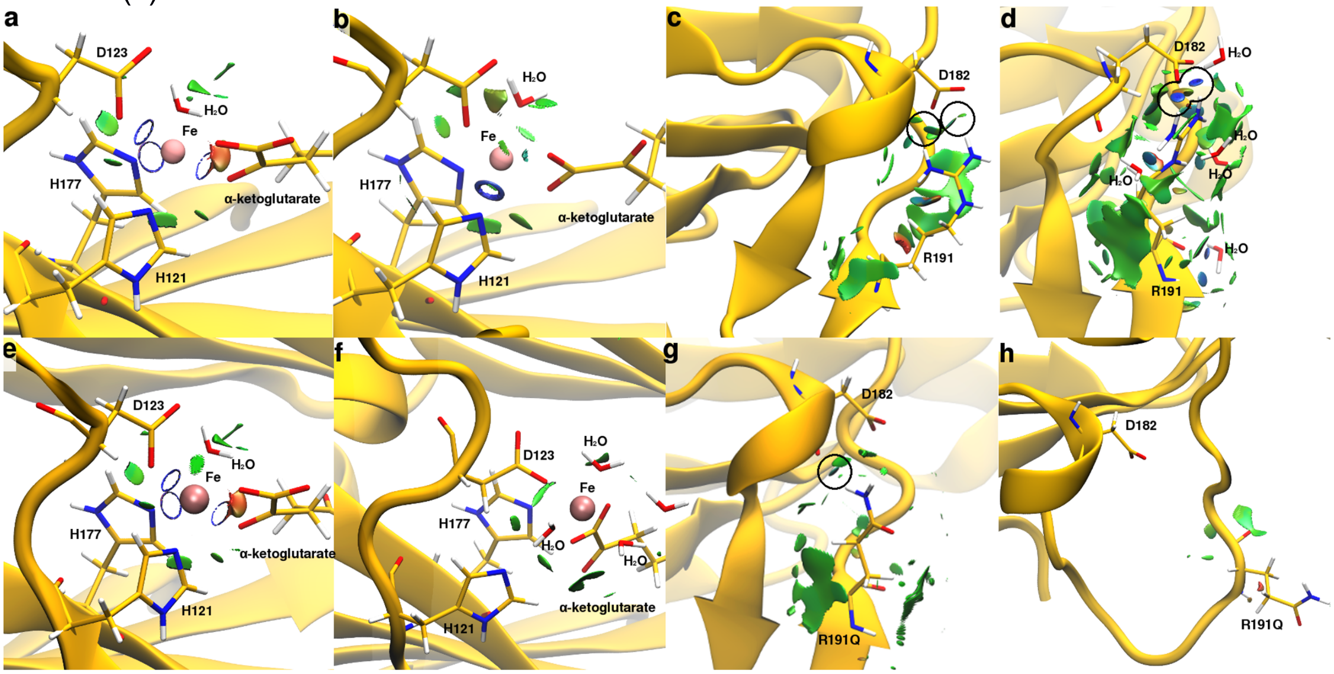

An animation for this transition for a representative mutant trajectory with succinate cofactors and using the NCI analysis is also available as electronic supporting information in the journal webpage.

S5 Figure: Average binding enthalpies for product succinate and Fe at the active site (in kcal/mol) over 500ns.

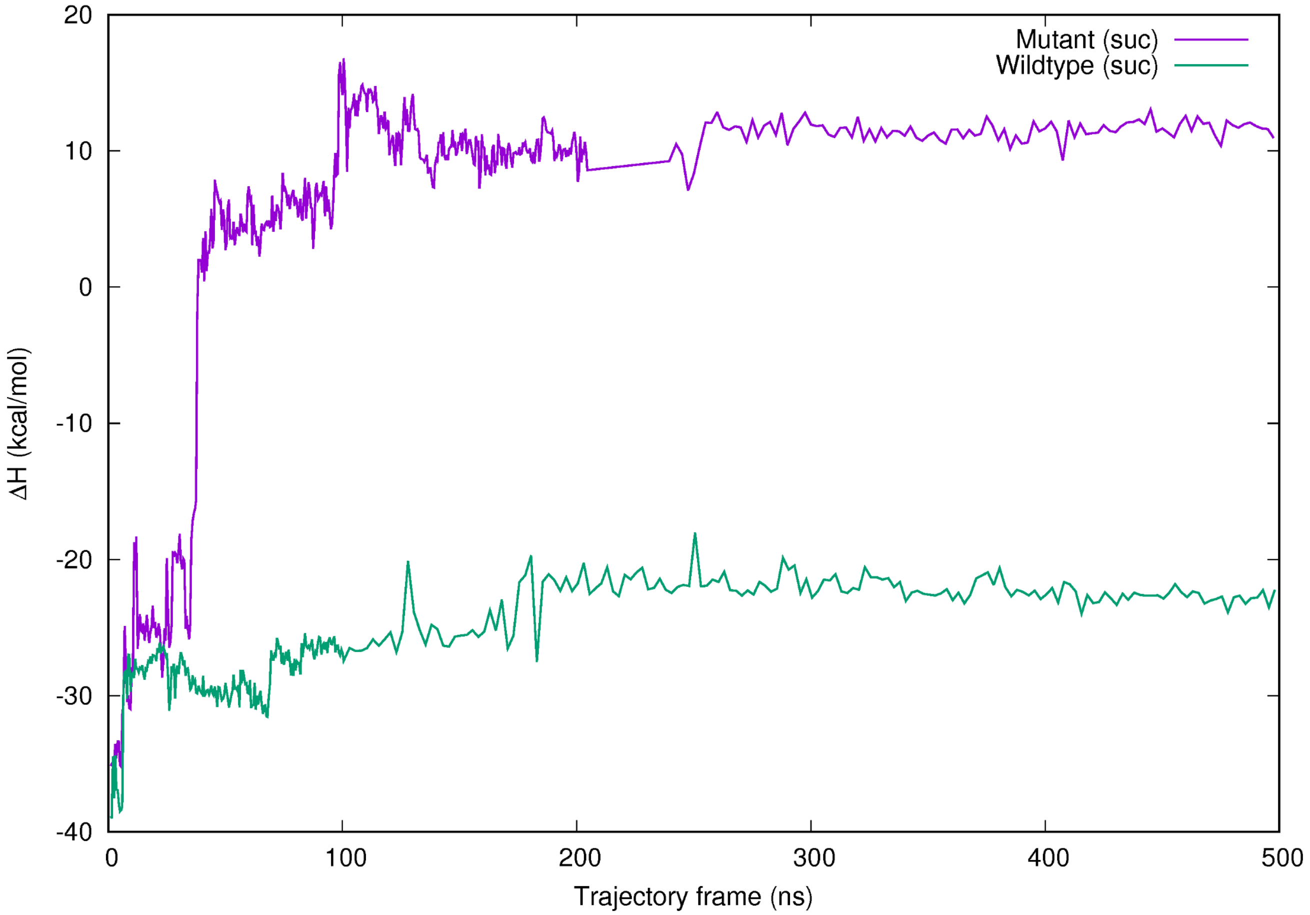

S3 Table: Average change in binding affinities between the duplicate trajectories. Energy for the metal-cofactor complex before and after the conformational shift (ΔG_{shift}), average free energy of binding for the cofactor-metal complex ($\Delta G_{binding}$), average change in binding enthalpy for the metal-cofactor complex before and after the conformational shift (ΔH_{shift}) and the average enthalpy of binding for the cofactor-metal complex ($\Delta H_{binding}$) between the duplicate trajectories. All energies are listed in kcal/mol.

Trajectory type	ΔG_{shift}	$\Delta G_{binding}$*	ΔH_{shift}	$\Delta H_{binding}$
Wild type	1.83±4.13	1.26±4.07	1.57±3.65	-29.20±3.85
Mutant	29.75±3.30	30.21±2.60	31.37±2.60	0.73±3.07

*These averages are taken subsequent to the conformational change (~100 ns) for the mutant trajectories.

On average, the standard deviation for each individual binding free energy calculation is around 4 kcal/mol--the standard deviation between the duplicate trajectories for the relative binding energies is similar, as can be seen in Table S2. Additionally, ΔH_{shift} and ΔG_{shift} are very similar, though the $\Delta G_{binding}$ and the $\Delta H_{binding}$ are substantially different, indicating that the change in the binding energy is likely enthalpically rather than entropically driven.

S6 Figure: SDS-PAGE analysis of ALKBH7. His-tagged WT ALKBH7 and its R191Q variant were purified by using a Ni-NTA Sepharose column, treated with TEV protease to remove the His-tag, and rechromatographed on the Ni-NTA Sepharose column to obtain the non-tagged proteins. The purified and concentrated proteins were analyzed by SDS-PAGE. Lane 1, WT ALKBH7; lane 2, R191Q ALKBH7; lane 3, His-tagged R191Q ALKBH7.

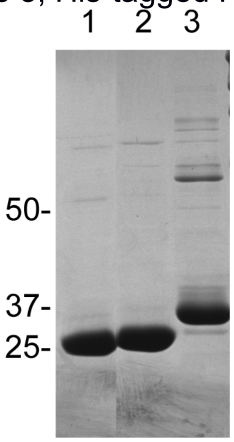

References

1. Contreras-García J, Johnson ER, Keinan S, Chaudret R, Piquemal JP, Beratan DN, et al. NCIPLOT: a program for plotting non-covalent interaction regions. J Chem Theo Comp. 2011; *7*, 625.
2. Wang G, He Q, Feng C, Liu Y, Deng Z, Qi X, et al. The atomic resolution structure of human AlkB homolog 7 (ALKBH7), a key protein for programmed necrosis and fat metabolism. J Biol Chem. 2014; *289*, 27924.
3. Blommel PG, Fox BG. A combined approach to improving large-scale production of tobacco etch virus protease. Prot Expr and Purif. 2007; *55*, 53.
