# Supplementary figures and images for: ALKBH7 Variant Related to Prostate Cancer Exhibits Altered Substrate Binding
Source: PLoS Comput Biol. 2017 Feb 23;13(2):e1005345. doi: 10.1371/journal.pcbi.1005345 (PMC5322872; doi:10.1371/journal.pcbi.1005345)

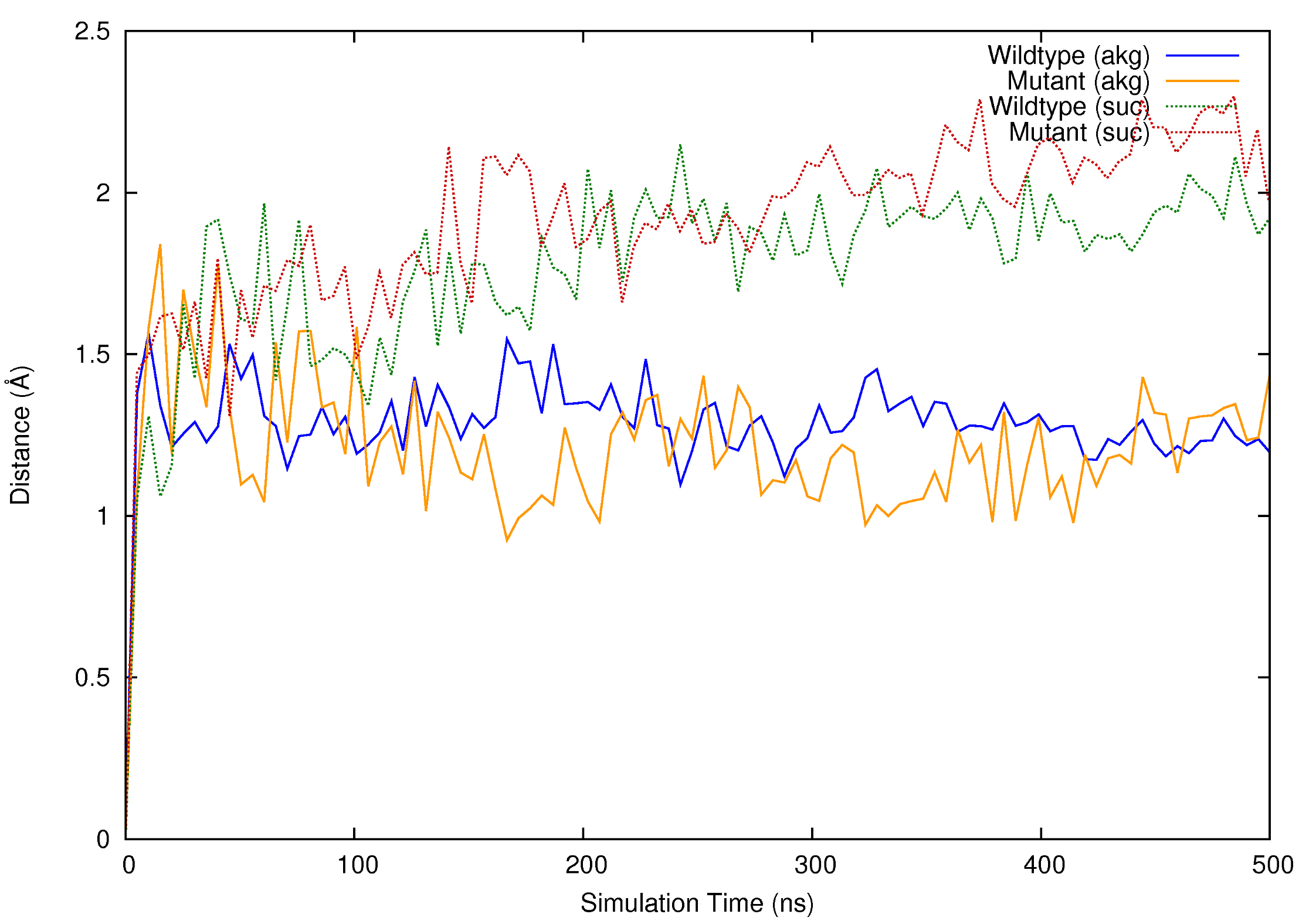

Supplement: S1 Fig — (TIF) [file pcbi.1005345.s002.tif]

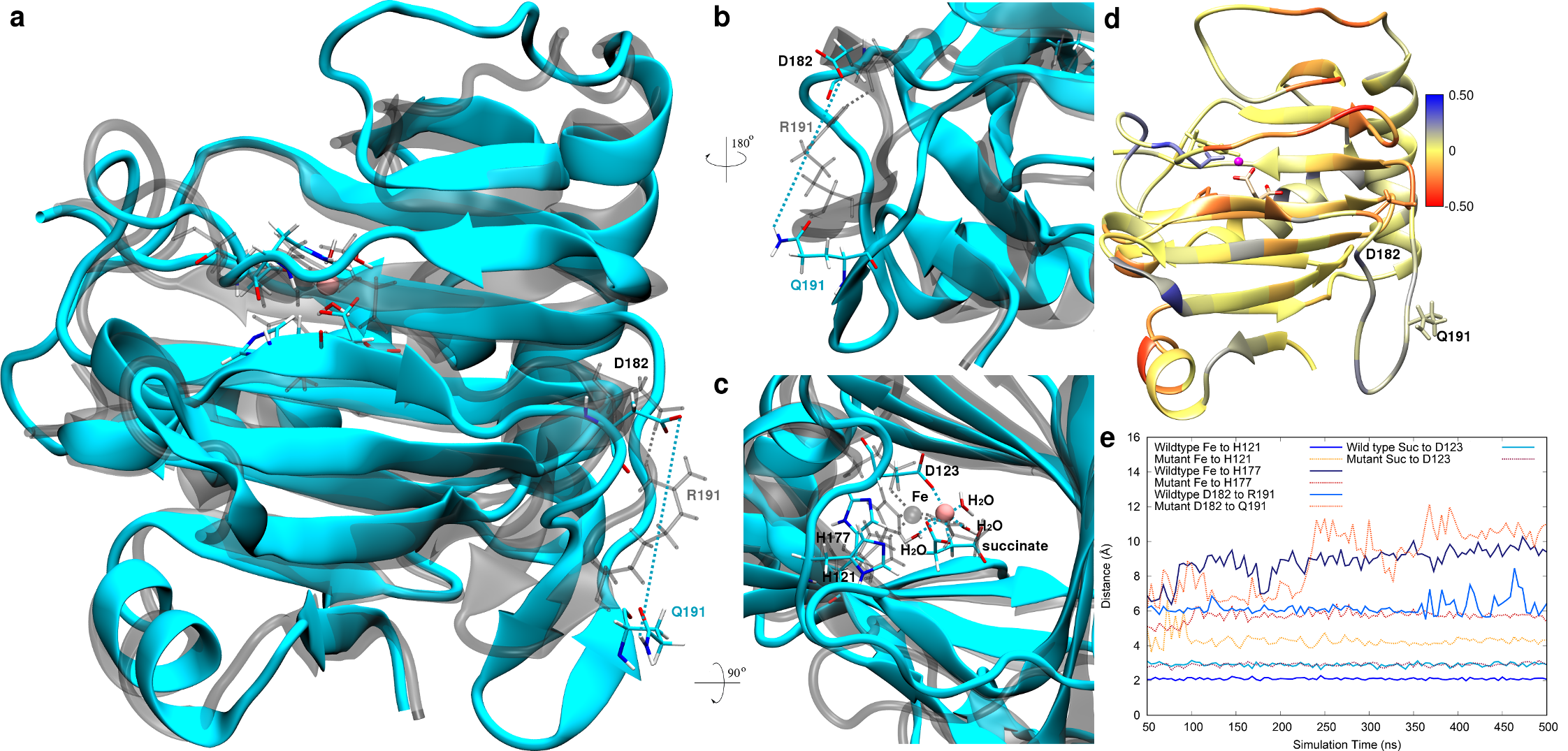

Supplement: S2 Fig — a, Overlay of representative structures for WT (gray) and R191Q variant (blue) forms of ALKBH7. Active site residues and succinate as well as the site undergoing substitution are displayed (licorice). b, 180 degree rotation and close-up of the substituted site. c, 90 degree rotation and close-up of the active site, with each relevant active site residue and succinate labeled. Dashed lines in gray represent the original bonds to the metal ion in the crystal structure, and dashed lines in blue represent the new bonds to the metal ion near the end of the trajectory for the mutant protein. d, Correlation difference for each residue in the WT protein with respect to the R191Q variant mapped onto the protein structure using the substituted site as the reference. e, Distance analysis for key residues in the mutation and active sites (with respect to their centers of mass) throughout the simulation trajectory. (TIF) [file pcbi.1005345.s003.tif]

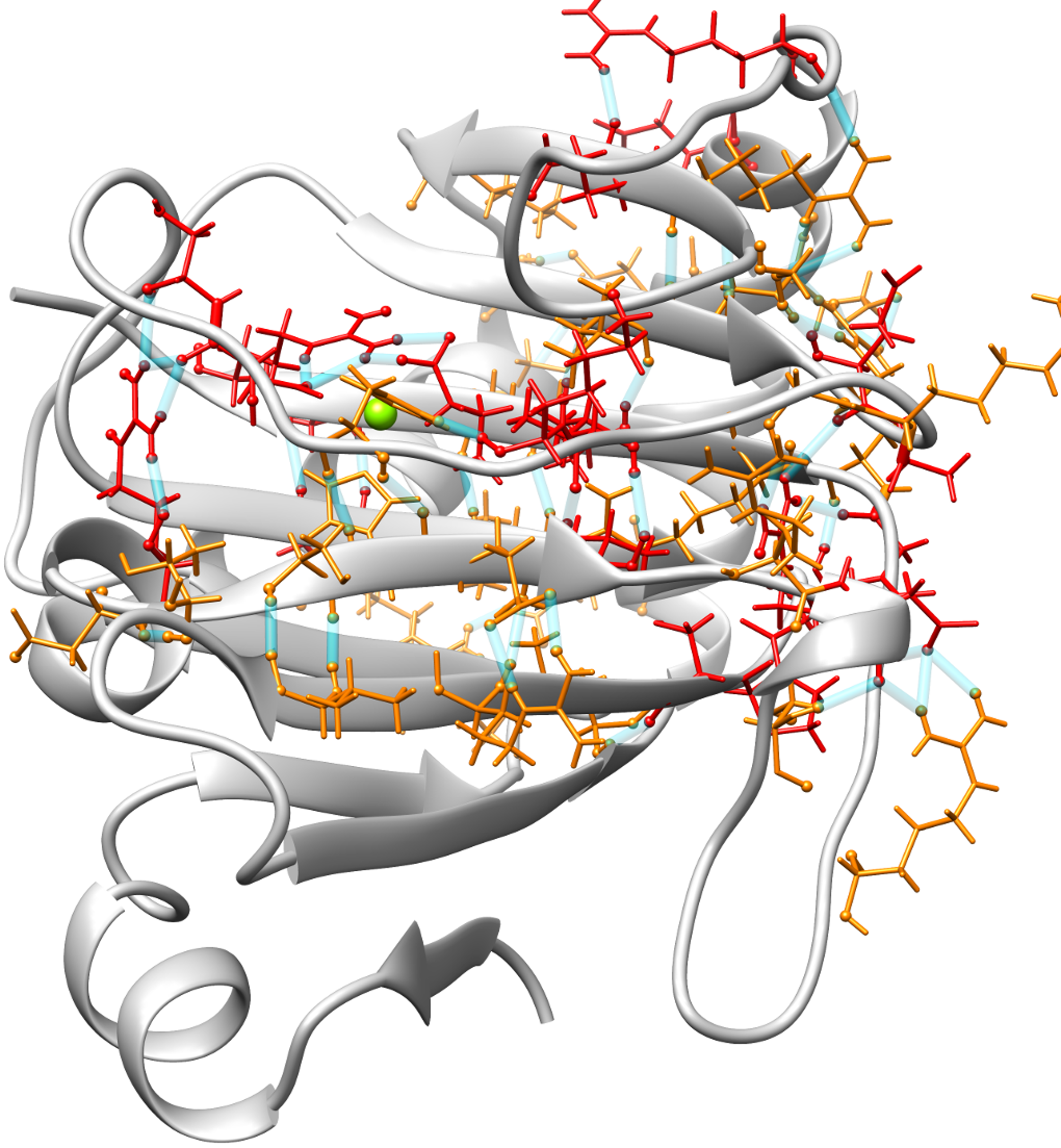

Supplement: S3 Fig — Residues colored in red denote amino acids involved in H-bonds for over 30% of the WT trajectory and broken for over 90% of the R191Q variant trajectory. Residues colored in orange are involved in hydrogen bonds for both trajectories, but are present for at least 30% less of the time in the variant trajectory. The hydrogen bonds between these residues are displayed in blue. This figure is for the WT/R191Q variant with succinate. (TIF) [file pcbi.1005345.s004.tif]

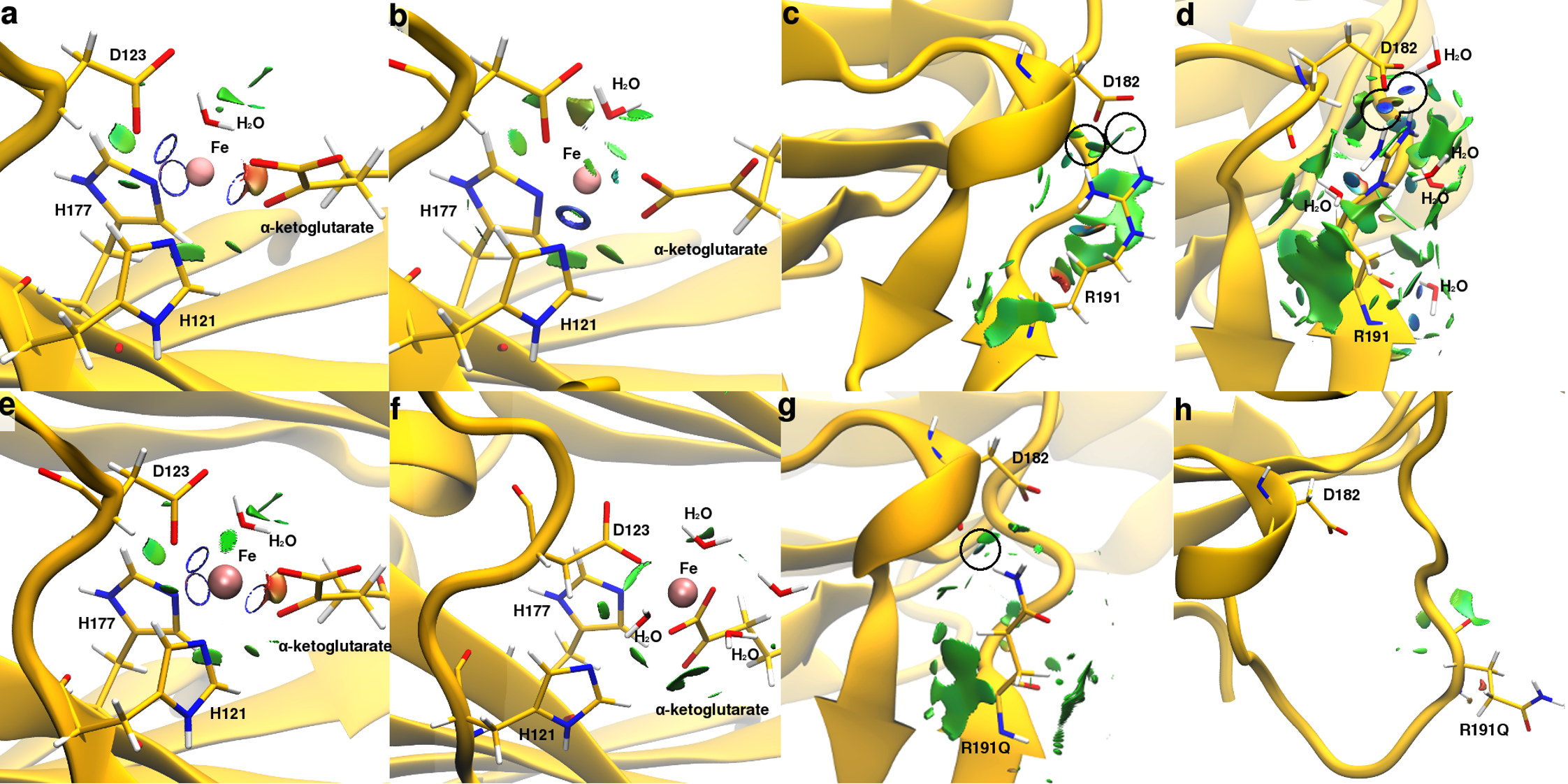

Supplement: S4 Fig — NCI plots of WT (a-d) and R191Q variant (e-h) ALKBH7. Panels show representative structures at different stages of the simulation showing the points prior to (a and e), during (b, c, f, and g) and after (d and h) the structural transition. The H-bonds between R191 and D182 in the WT structure that are removed in the SNP variant are circled in black (c). (TIF) [file pcbi.1005345.s005.tif]

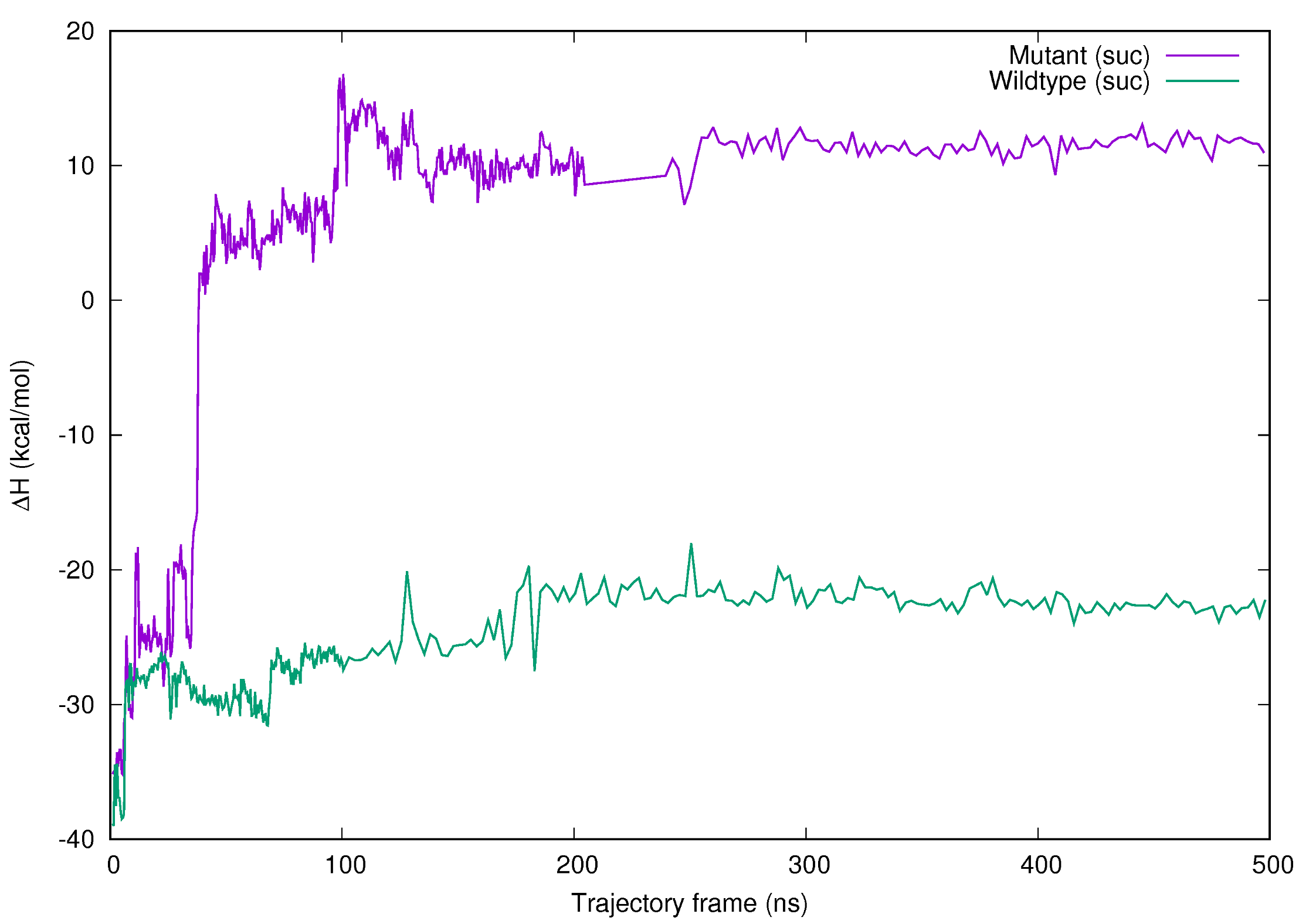

Supplement: S5 Fig — (TIF) [file pcbi.1005345.s006.tif]

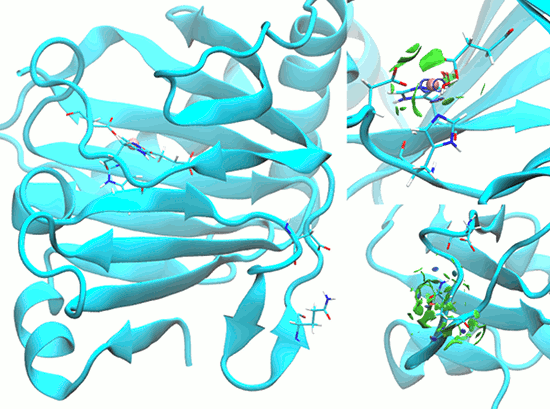

Supplement: S1 Video — Each panel has NCIplot surfaces to demonstrate the change in the intermolecular forces, updated at key points along the animation. (GIF) [file pcbi.1005345.s008.gif]
