# Supplementary material for: ALKBH7 Variant Related to Prostate Cancer Exhibits Altered Substrate Binding
Source: PLoS Comput Biol. 2017 Feb 23;13(2):e1005345. doi: 10.1371/journal.pcbi.1005345 (PMC5322872; doi:10.1371/journal.pcbi.1005345)
Supplement: S1 Table — (DOCX) [file pcbi.1005345.s009.docx]

**Table S1. SNPs with significant association to a prostate cancer phenotype**

| **#** | **SNP** | **Gene** | **p-value** | **Intron/ Exon** | **Effects of the Missense Mutation** |
| --- | --- | --- | --- | --- | --- |
|  |  |  |  |  |  |
|  | **Multiplicative Model** | | |  |  |
| 1 | rs7160307 | *ALKBH1* | 0.00754 | I |  |
| 2 | rs3751812 | *FTO* | 0.04365 | I |  |
| 3 | rs6499653 | *FTO* | 0.029584 | I |  |
| 4 | rs8044769 | *FTO* | 0.048004 | I |  |
|  |  |  |  |  |  |
|  | **Recessive Model** | |  |  |  |
| 1 | rs7160307 | *ALKBH1* | 0.044301 | I |  |
| 2 | rs7540 | *ALKBH7* | 0.029142 | E | Arg to Gln |
| 3 | rs6499653 | *FTO* | 0.009707 | I |  |
| 4 | rs7193938 | *FTO* | 0.020805 | I |  |
| 5 | rs8044769 | *FTO* | 0.014301 | I |  |
| 6 | rs9302652 | *FTO* | 0.034896 | I |  |
|  |  |  |  |  |  |
|  | **Additive Model** | |  |  |  |
| 1 | rs7160307 | *ALKBH1* | 0.012976 | I |  |
| 2 | rs7540 | *ALKBH7* | 0.042607 | E | Arg to Gln |
| 3 | rs3751812 | *FTO* | 0.018385 | I |  |
| 4 | rs6499653 | *FTO* | 0.00888 | I |  |
| 5 | rs7190492 | *FTO* | 0.035297 | I |  |
| 6 | rs7193938 | *FTO* | 0.038663 | I |  |
| 7 | rs8044769 | *FTO* | 0.030062 | I |  |
| 8 | rs8050136 | *FTO* | 0.022015 | I |  |
| 9 | rs9302652 | *FTO* | 0.031647 | I |  |
|  |  |  |  |  |  |
|  | **Dominant Model** | |  |  |  |
| 1 | rs7160307 | *ALKBH1* | 0.006068 | I |  |
| 2 | rs3751812 | *FTO* | 0.019815 | I |  |
| 3 | rs8050136 | *FTO* | 0.022225 | I |  |
| 4 | rs12447481 | *FTO* | 0.033246 | I |  |
