# Supplementary material for: ALKBH7 Variant Related to Prostate Cancer Exhibits Altered Substrate Binding
Source: PLoS Comput Biol. 2017 Feb 23;13(2):e1005345. doi: 10.1371/journal.pcbi.1005345 (PMC5322872; doi:10.1371/journal.pcbi.1005345)
Supplement: S2 Table — The average distances, RMSD of the protein backbone, and the total number of hydrogen bonds over the entirety of each trajectory were calculated individually and then averaged together to obtain the average and standard deviation values across trajectories so that the replicate runs could be compared. (DOCX) [file pcbi.1005345.s010.docx]

**Table S2: Average distances and structural details across all duplicate trajectories.** The average distances, RMSD of the protein backbone, and the total number of hydrogen bonds over the entirety of each trajectory were calculated individually and then averaged together to obtain the average and standard deviation values across trajectories so that the replicate runs could be compared.

| Trajectory type | Fe to H121(Å) | Fe to H177(Å) | D182 to R191/Q191(Å) | Suc or akg to D123(Å) | Backbone RMSD (Å) | Total # hydrogen bonds |
| --- | --- | --- | --- | --- | --- | --- |
| Wild type | 2.10±0.05 | 4.05±2.81 | 6.30±0.39 | 3.02±0.27 | 1.52±0.20 | 112±4 |
| Mutant | 4.14±0.03 | 5.59±0.46 | 8.03±0.99 | 2.82±0.10 | 1.68±0.44 | 107±3 |
