# Supplementary material for: ALKBH7 Variant Related to Prostate Cancer Exhibits Altered Substrate Binding
Source: PLoS Comput Biol. 2017 Feb 23;13(2):e1005345. doi: 10.1371/journal.pcbi.1005345 (PMC5322872; doi:10.1371/journal.pcbi.1005345)
Supplement: S3 Table — Energy for the metal-cofactor complex before and after the conformational shift (ΔGshift), average free energy of binding for the cofactor-metal complex (ΔGbinding), average change in binding enthalpy for the metal-cofactor complex before and after the conformational shift (ΔHshift) and the average enthalpy of binding for the cofactor-metal complex (ΔHbinding) between the duplicate trajectories. All energies are listed in kcal/mol. (DOCX) [file pcbi.1005345.s011.docx]

**Table S3: Average change in binding affinities between the duplicate trajectories.** Energy for the metal-cofactor complex before and after the conformational shift (Δ*G_shift_)*, average free energy of binding for the cofactor-metal complex (Δ*G_binding_),* average change in binding enthalpy for the metal-cofactor complex before and after the conformational shift (ΔH*_shift_*) and the average enthalpy of binding for the cofactor-metal complex (Δ*H_binding_*) between the duplicate trajectories. All energies are listed in kcal/mol.

| Trajectory type | Δ*G_shift_* | Δ*G_binding_** | ΔH*_shift_* | Δ*H_binding_* |
| --- | --- | --- | --- | --- |
| Wild type | 1.83±4.13 | 1.26±4.07 | 1.57±3.65 | -29.20±3.85 |
| Mutant | 29.75±3.30 | 30.21±2.60 | 31.37±2.60 | 0.73±3.07 |

*These averages are taken subsequent to the conformational change (~100 ns) for the mutant trajectories.
